# Advising overweight persons about diet and physical activity in primary health care: Lithuanian health behaviour monitoring study

**DOI:** 10.1186/1471-2458-6-30

**Published:** 2006-02-14

**Authors:** Jurate Klumbiene, Janina Petkeviciene, Vytautas Vaisvalavicius, Irena Miseviciene

**Affiliations:** 1 Institute for Biomedical Research, Kaunas University of Medicine, Eiveniu str. 4, 50009 Kaunas 7, Lithuania

## Abstract

**Background:**

Obesity is a globally spreading health problem. Behavioural interventions aimed at modifying dietary habits and physical activity patterns are essential in prevention and management of obesity. General practitioners (GP) have a unique opportunity to counsel overweight patients on weight control. The purpose of the study was to assess the level of giving advice on diet and physical activity by GPs using the data of Lithuanian health behaviour monitoring among adult population.

**Methods:**

Data from cross-sectional postal surveys of 2000, 2002 and 2004 were analysed. Nationally representative random samples were drawn from the population register. Each sample consisted of 3000 persons aged 20–64 years. The response rates were 74.4% in 2000, 63.4% in 2002 and 61.7% in 2004. Self-reported body weight and height were used to calculate body mass index (BMI). Information on advising in primary health care was obtained asking whether GP advised overweight patients to change dietary habits and to increase physical activity. The odds of receiving advice on diet and physical activity were calculated using multiple logistic regression analyses according to a range of sociodemographic variables, perceived health, number of visits to GPs and body-weight status.

**Results:**

Almost a half of respondents were overweight or obese. Only one fourth of respondents reported that they were advised to change diet. The proportion of persons who received advice on physical activity was even lower. The odds of receiving advice increased with age. A strong association was found between perceived health and receiving advice. The likelihood of receiving advice was related to BMI. GPs were more likely to give advice when BMI was high. More than a half of obese respondents (63.3%) reported that they had tried to lose weight. The association between receiving advice and self-reported attempt to lose weight was found.

**Conclusion:**

The low rate of dietary and physical activity advice reported by overweight patients implies that more lifestyle counselling should be provided in primary health care. There is an obvious need for improved training and education of GPs in counselling of overweight patients focusing on methods of giving dietary and physical activity advice.

## Background

Obesity is a globally spreading health problem. Diabetes, hypertension, dyslipidaemia, cardiovascular disease, and some cancers are associated with obesity [1,2]. Those co-morbidities have considerable health care and social cost [3,4]. The World Health Organisation report on obesity states that sedentary lifestyle and consumption of high-fat energy-dense diets are fundamental causes of the obesity epidemic [1]. Health promotion strategies, including behavioural interventions aimed at modifying dietary habits and physical activity patterns are essential in prevention and management of obesity.

Primary health care has a unique opportunity for health promotion activities. A substantial part (60–70%) of population makes visits to their general practitioner (GP) each year [5]. There is sufficient evidence that the majority of people regard doctors as the best and most credible source of advice on a range of issues, including diet and physical activity [6,7]. However, the studies have shown low rate of counselling on lifestyle changes given to overweight patients in primary health care [8,9].

Prevalence of overweight and obesity is high in Lithuania [10]. Every tenth adult is obese and every third has overweight. Effective management strategies of overweight require common efforts of health care services and community.

Primary health care as a separately organised sector of health services is a new concept in Lithuania. In 1995, the Ministry of Health approved the establishment of GP institution and defined its role. Programmes of training and retraining of GPs were started. Lithuanian regulation "The GP norm" specifies GPs' activities, including counselling on weight control. However, there is a lack of data how often GPs are giving advice to obese patients.

This study is aimed at assessing the level of giving advice on diet and physical activity by GPs using the data of Lithuanian health behaviour monitoring among adult population within the framework of the international FINBALT HEALTH MONITOR project [11].

## Methods

Data from cross-sectional postal surveys of 2000, 2002 and 2004 were used. Nationally representative random samples were drawn from the population register. The sampling unit was an individual in all the surveys and no measures were taken to substitute for non-respondents. Each sample consisted of 3000 persons aged 20–64 years. The questionnaires were mailed in April and one reminder was sent. The response rates were 74.4% in 2000, 63.4% in 2002 and 61.7% in 2004.

We analysed the data of respondents who visited their GP within the previous 12 months. The number of persons visiting a general practitioner was 1497 (68.2% of all respondents) in 2000, 1407 (74.7%) in 2002 and 1384 (77.3%) in 2004.

Self-reported body weight and height were used to calculate body mass index (BMI), defined as weight in kilograms divided by the square of height in meters. BMI was categorised into four groups: of normal weight (BMI < 25 kg/m^2^), overweight (BMI – 25–29 kg/m^2^), obese (BMI – 30–34 kg/m^2^) and severe obese (BMI ≥ 35 kg/m^2^). The data of overweight and obese persons were included into the analysis.

Information on advising in primary health care was obtained asking the following questions: 'During the last year (12 months) have you been advised to change your dietary habits?' and 'During the last year (12 months) have you been advised to increase your physical activity?'

Education was measured by three educational levels: incomplete secondary, secondary and university. The respondents were grouped according to their place of residence as living in cities, towns or villages. Marital status was dichotomised as 'married' and 'others'. Information on perceived health was elicited by the following question 'How would you assess your present state of health?: 1) Good, 2) reasonably good, 3) average, 4) rather poor, 5) poor'. It was categorised as 'good' (1+2), 'average' (3) and 'poor' (4+5) (Table 1). People were asked how often they had seen a GP during the previous 12 months. They were grouped as visiting 1–2 times, 3–4 times, and 5 times or more.

Data were analysed using the statistical package SPSS version 12.1. One database was compiled from the three surveys and it included the corresponding variables described. The differences in the distribution of respondents by body-weight status were assessed using analyses of chi-squared tests. The odds of receiving advice on diet and physical activity were calculated using multiple logistic regression analyses according to a range of sociodemographic variables, perceived health, number of visits to GPs and body-weight status. The first category of each factor was the reference category. When the 95% confidence interval did not include 1, the odds ratio was considered to be statistically significant.

The investigation was conformed to the principles outlined in the Declaration of Helsinki and approved by the regional ethics committee.

## Results

The distribution of men and women according to BMI is shown in Table 2. Almost a half of respondents were overweight or obese. The prevalence of overweight and obesity increased with age both in men and women.

GPs were not very active in advising overweight persons to change their dietary habits and to increase physical activity. Only one fourth of respondents reported that they were told to change diet (Table 3). The proportion of persons who received advice on physical activity was even lower. The odds of receiving advice increased with age. The reported receipt of advice was not related to gender, education and place of residence. A strong association was found between perceived health and receiving advice. Persons with poor health were five times as likely to be advised as those with good health. The proportion of persons who reported that they had been advised increased with the increasing number of visits to a GP. The likelihood of receiving advice was related to BMI. GPs were more likely to give advice when BMI was high.

More than a half of obese respondents (63.3%) reported that they had tried to lose weight. More women than men reported attempts to reduce weight. The association between receiving advice and self-reported attempt to lose weight was found. Men and women being advised to increase physical activity and women being advised to change diet were more likely to make attempts to reduce weight (Table 4).

## Discussion

Our findings show that the proportion of the overweight respondents who had visited general practitioner during the past 12 months and received advice for losing weight was small. The receipt of advice was associated with age, perceived health, number of visits to GP and BMI. Respondents who were advised more often reported attempts to lose weight than those who were not advised.

Our study has several limitations. The overweight and obesity were assessed using self-reported data on weight and height. Overweight persons are linked to underreport their weight. Therefore our sample could not included marginally overweight persons who possibly are less often advised than the obese ones. Another obvious limitation lies in the assessment of GPs activities based on patient's reports. People may underestimate the frequency of receiving advice.

Other studies that evaluated counselling obese patients by GPs have shown that proportion of advised persons varies from 5% to more than 40% [8,9,12,13]. Several barriers in advising patients were emphasised by researchers. The patient's lack of motivation or will to make the required lifestyle changes is regarded as the main barrier. The study undertaken by the European Network for Prevention and Health Promotion in Family Medicine and General Practice (EUROPREV) demonstrated that more than half of GPs were sceptical about helping patients achieve or maintain normal weight [14]. In Canada 48% of GPs considered that dietary change had little effect on weight control [15].

GPs were more likely to provide advice to patients they believe most likely to change unhealthy behaviour [12,16]. Consistent with findings of previous studies, GPs in Lithuania tended to advise those who were most overweight or might have some health problems. Persons who frequently visited GP received advice more often than those who had check-up only once or twice a year.

Time resources in primary health care are limited. Most GPs mentioned that one of the constraints to offering advice was a lack of time [14,17]. If GP spends time on advice this will reduce the time for the rest of the consultation.

Several studies have shown that GPs knowledge about nutrition and physical activity in management of obesity is incomplete [8,18]. They expressed the need for clinical guidelines and supplementary training. Even when GPs have nutritional knowledge, they find it difficult to communicate this knowledge effectively. GPs would therefore benefit from additional training in counselling skills.

The studies have reported that education and medical advice to lose weight were strongly associated with trying to reduce weight [12,13,19]. The same association was found in our study. Women appear to be more likely than men to be engaged in attempts to lose weight. One reason for such difference could be that women are more dissatisfied with their body shape compared with men [20]. Being thin is a desirable body ideal for women. They more often than men choose food they considered to be healthy while men more often prefer food they like [21].

In our study, overweight and obesity were estimated by using BMI which alone is not a sufficient predictor of metabolic abnormalities. There is more and more evidence that waist circumference is associated with all-cause mortality and risk of coronary heart disease [22,23]. Because weight loss can reduce risk factors for chronic diseases, appropriated determination of height, weight and waist circumference should be carried out in primary health care settings and health care professionals need to be more active in advising of obese patients on diet and physical activity. However, many doctors practising today in Lithuania have never been taught about counselling of obese patients.

## Conclusion

The low rate of dietary and physical activity advice reported by overweight patients implies that more lifestyle counselling should be provided in primary health care. There is an obvious need for improved training and education of GPs in counselling of overweight patients focusing on methods of giving dietary and physical activity advice.

## Competing interests

The author(s) declare that they have no competing interests.

## Authors' contributions

JK designed and managed the study, supervised whole process, revised the draft. JP formulated the general research question, participated in the design of the study, advised on the interpretation of the results. VV performed statistical analysis and drafted the manuscript. IM provided administrative support, critically reviewed draft. All authors have read and approved the final manuscript.

## Pre-publication history

The pre-publication history for this paper can be accessed here:



## Figures and Tables

**Table 1:** Characteristics of overweight and obese persons who visited their general practitioner within the previous 12 months

Characteristics	n	%
*Total*	2049	100
*Gender*		
Men	893	43.6
Women	1156	56.4
*Age group (years)*		
20–34	333	16.2
35–49	842	41.1
50–64	874	42.7
*Education group*		
Incomplete secondary	277	13.5
Secondary	1321	64.5
University	451	22.0
*Place of residence*		
Cities	900	43.9
Towns	660	32.2
Villages	489	23.9
*Marital status*		
Married	1545	75.4
Unmarried	504	24.6
*Perceived health status*		
Good	618	30.2
Average	1165	56.8
Poor	266	13.0
*Visits to GP during the last yea*r		
1–2 times	985	48.1
3–4 times	390	19.0
5 times and more	674	32.9

**Table 2:** Distribution (%) of respondents by the categories of body mass index (BMI)

BMI (kg/m^2^)	Men				Women			
	
	Age				Age			
	
	20–34	35–49	50–64	Total	20–34	35–49	50–64	Total
<25	65.8	40.5	33.3	46.3	82.2	51.1	28.3	54.8
25–29.99	28.5	45.3	41.1	38.7	13.1	33.2	38.2	28
30–34.99	4.7	12.1	18.2	11.6	3.2	11.4	23.1	12.1
≥35	1.0	2.1	7.4	3.4	1.5	4.3	10.4	5.1
Total	100	100	100	100	100	100	100	100

P < 0.001 for differences between age groups both in men and women based on χ^2 ^test

**Table 3:** Factors associated with receiving advice from a general practitioner (GP) among overweight and obese persons who visited their general practitioner within the previous 12 months

Characteristic	Persons receiving advice to change dietary habits (%)	Adjusted OR* (95%CI)	Persons receiving advice to increase physical activity (%)	Adjusted OR* (95%CI)
*Sex*				
Men	22.5	1.00	15.9	1.00
Women	29.4	1.17(0.93–1.46)	19.2	1.02(0.63–1.65)
*Age group (years)*				
20–34	10.9	1.00	3.5	1.00
35–49	23.7	1.79(1.19–2.68)	15.2	4.35(1.42–13.31)
50–64	34.7	2.18(1.45–3.27)	26.1	5.34(1.76–16.23)
*Education group*				
Incomplete secondary	31.8	1.00	18.3	1.00
Secondary	25.3	1.06(0.77–1.47)	17.0	1.30(0.64–2.66)
University	27.0	1.40(0.96–2.04)	20.1	2.26(0.99–5.17)
*Place of residence*				
Cities	26.0	1.00	19.0	1.00
Towns	27.6	1.01(0.79–1.29)	17.4	0.74(0.44–1.27)
Villages	25.6	0.81(0.61–1.07)	16.7	0.71(0.39–1.30)
*Marital status*				
Married	26.3	1.00	18.4	1.00
Unmarried	26.9	0.90(0.70–1.16)	15.6	0.91(0.51–1.62)
*Perceived health status*				
Good	11.3	1.00	6.2	1.00
Average	29.5	2.61(1.93–3.52)	20.5	2.80(1.37–5.71)
Poor	48.5	5.32(3.62–7.82)	31.8	4.77(2.03–11.21)
Visits to GP during the last year				
1–2 times	12.8	1.00	6.0	1.00
3–4 times	28.4	2.36(1.74–3.21)	11.2	1.75(1.14–2.68)
5 times and more	45.2	3.88(2.97–5.08)	22.2	3.22(2.25–4.59)
*Body mass index kg/m^2^*				
25–29.99	19.0	1.00	8.6	1.00
30–34.99	36.4	2.09(1.65–2.66)	28.3	4.49(2.68–7.51)
≥35	52.9	3.82(2.72–5.35)	52.2	11.36(6.03–21.43)

* Adjusted for all variables included in the model

**Table 4:** Proportion (%) of obese persons who tried to reduce the weight in relation to receiving advice

	Men			Women		
	
	%	95% CI	P	%	95% CI	P
*Advised to change dietary habits*						
Yes	58.8	42.3–75.3	0,11	86.0	76.4–95.6	0,01
No	42.0	30.4–53.6		67.6	59.0–76.2	
*Advised to increase physical activity*						
Yes	78.9	60.6–97.2	0,002	85.7	72.7–98.7	0,10
No	40.5	30.0–51.0		70.6	62.9–72.3	

CI – confidence intervals
